# Breast Cancer Awareness and Screening Predictors in Young Saudi Women: Findings From a Cross‐Sectional Study

**DOI:** 10.1002/hsr2.71831

**Published:** 2026-02-24

**Authors:** Jobran M. Moshi, Siddig Ibrahim Abdelwahab, Manal Mohamed Elhassan Taha, Dhuha A. Alamer, Haitham M. Maashi, Raya M. Badri, Mohammed Jeraiby, Sama M. Maashi, Ebhar Jubran M. Majrashi, Yazan A. Bajawi, Humaid Obaid Al‐Shamsi

**Affiliations:** ^1^ Department of Medical Laboratory Technology, College of Nursing and Health Science Jazan University Jazan Kingdom of Saudi Arabia; ^2^ Health Research Centre Jazan University Jazan Saudi Arabia; ^3^ Jazan Education Department Ministry of Education Jazan Kingdom of Saudi Arabia; ^4^ Faculty of Medicine Jazan University Jazan Kingdom of Saudi Arabia; ^5^ Burjeel Cancer Institute, Burjeel Medical City Abu Dhabi UAE; ^6^ Department of Medical Oncology Harvard Medical School, Dana‐Farber Cancer Institute Boston Massachusetts USA; ^7^ College of Medicine Ras Al Khaimah Medical and Health Sciences University Ras Al Khaimah UAE; ^8^ Gulf Medical University Ajman UAE; ^9^ Emirates Oncology Society Dubai UAE; ^10^ College of Medicine University of Sharjah Sharjah UAE

**Keywords:** awareness, breast cancer, breast self‐examination, early detection, Saudi Arabia, screening behavior

## Abstract

**Background and Aims:**

Breast cancer is the most commonly diagnosed cancer among women in Saudi Arabia, with early detection playing a critical role in improving survival outcomes. However, screening uptake remains suboptimal, particularly among younger women. To address this gap, this study aimed to evaluate the knowledge, attitudes, and practices (KAP) of young women in the Jazan region and identify key demographic and behavioral predictors influencing breast cancer screening uptake.

**Methods:**

A cross‐sectional study (*N* = 480) was conducted between January and March 2024. A structured, validated questionnaire was used to collect data on demographic characteristics and KAP regarding breast cancer. The primary outcomes assessed were knowledge, attitude, and practice scores. Statistical analysis included descriptive and inferential statistics, Pearson's correlation, and logistic regression to identify predictors of breast self‐examination (BSE).

**Results:**

Most participants were young, single Saudi students residing in urban areas. The mean scores were: knowledge (3.66 ± 0.66), attitude (4.09 ± 0.58), and practice (3.81 ± 0.73). Moderate‐to‐strong positive correlations were observed between knowledge and attitude (*r* = 0.569, *p* < 0.001) and between knowledge and practice (*r* = 0.567, *p* < 0.001), indicating meaningful associations among the three domains. Age differences were evident, with both the youngest (< 25 years) and oldest (> 45 years) groups demonstrating comparatively higher knowledge scores. Participants in health‐related specializations also showed higher knowledge levels (mean = 3.78 ± 0.66), suggesting a notable effect of academic background. Logistic regression demonstrated that age (26–35 years) substantially increased the likelihood of performing BSE (adjusted OR = 12.422; 95% CI: 1.12–137.95), while having two to three children markedly reduced the odds (adjusted ORs = 0.051–0.065). Additionally, non‐Saudi participants had lower odds of practicing BSE (crude OR = 0.212; 95% CI: 0.06–0.72), highlighting demographic disparities in screening behaviors.

**Conclusion:**

Age, education, specialization, and family responsibilities significantly influence women's breast cancer knowledge, attitudes, and preventive behaviors, including BSE and screening uptake. Targeted, population‐specific awareness strategies are essential to enhance early detection practices and reduce the breast cancer burden in the KSA.

## 1 Introduction

By 2020, breast cancer had emerged as the most frequently diagnosed cancer worldwide, surpassing lung and prostate cancers [1]. The global incidence continues to rise, with projections estimating that cases in the United States alone will reach 364,000 by 2040 [2]. While part of this increase may reflect improved detection, rising incidence in less developed nations is also linked to shifts in reproductive patterns and lifestyle behaviours [3]. In Saudi Arabia, breast cancer represents the most common cancer among women, accounting for 30%–31% of all female cancers, with incidence rising sharply in recent decades. National data indicate an age‐standardized incidence rate (ASR) of ~100.4/100,000 women (2022) and a mortality rate of 7.6/100,000 women (2020). Alarmingly, incidence rates have increased by more than 150% between 2001 and 2017, underscoring an escalating public health challenge in the Kingdom [4, 5, 6, 7, 8]. These statistics highlight the urgent need to strengthen early detection, awareness, and screening behaviors among Saudi women.

Breast cancer screening includes many methods, such as mammography, clinical breast examination (CBE), and breast self‐examination (BSE), designed to identify breast cancer in the early asymptomatic phases when therapy is the most efficacious [9]. Screening is essential for decreasing breast cancer mortality by facilitating prompt detection and management, thus enhancing treatment results and survival rates. Screening is commonly recommended as a fundamental component of breast cancer management methods because of its ability to detect malignant tumors prior to their clinical manifestation [10, 11, 12]. In Saudi Arabia, breast cancer is a substantial health burden, characterized by increasing incidence rates and related death [13, 14]. Despite initiatives to enhance breast cancer awareness and screening, the use of screening services among Saudi women remains inadequate. This occurrence is influenced by several variables, such as sociocultural norms, restricted access to healthcare facilities, insufficient information and awareness about breast cancer and screening methods, and fear or stigma linked to cancer diagnosis and treatment [15, 16, 17]. Consequently, numerous women forgo routine screenings, resulting in delayed diagnoses and worse treatment outcomes. It is essential to confront these obstacles to enhance screening participation, promote early diagnosis, and alleviate the impact of breast cancer in Saudi Arabia [13, 14].

Breast cancer screening uptake in Saudi Arabia remains low, with fewer than 25%–30% of women participating in regular screening. Although national initiatives—such as the Saudi National Breast Cancer Early Detection Program and widespread awareness campaigns—have increased public messaging, screening behaviors, including BSE and mammography, remain limited due to persistently low knowledge levels, cultural beliefs, fear, and misconceptions [18]. Previous studies have identified predictors such as age, education, marital status, number of children, and employment; however, important gaps remain, especially regarding younger women, who represent a growing but understudied population. Additionally, regional variations are rarely addressed, and data from the Jazan area are particularly scarce. Existing literature typically examines knowledge, attitudes, or practices separately, without assessing how these constructs interact to shape screening behavior [18, 19, 20]. Therefore, this study fills a significant gap by investigating the predictors of breast cancer knowledge, attitudes, and screening practices among young Saudi women in Jazan, with the goal of guiding more targeted and culturally sensitive interventions that enhance early detection behaviors.

## 2 Materials and Methods

### 2.1 Study Design and Setting

This cross‐sectional study was conducted in the Jazan area of southwestern Saudi Arabia from May to October 2024 using a random sample of literate women aged 18 years and older. Participants were recruited through community outreach channels and screened for eligibility based on age, literacy, and residence. Before participation, all individuals received a clear explanation of the study's purpose, procedures, risks, and confidentiality measures. Written informed consent was obtained electronically, and participation was voluntary. All data were anonymized, securely stored, and handled in accordance with institutional guidelines and the Declaration of Helsinki. The study was designed and reported in alignment with the Strengthening the Reporting of Observational Studies in Epidemiology (STROBE) guidelines [21].

### 2.2 Sampling and Recruitment

Data were gathered via a link, including a statement written by the study team, which was disseminated by the researchers and trained volunteers to ensure the integrity of the obtained data. The questions were reduced to become administrative obligatory for replies.

The sample size was determined using the standard formula for estimating a proportion in cross‐sectional studies [22, 23, 24, 25, 26, 27, 28, 29]:

$$n=Z^{2}\cdot p(1–p)/d^{2}.$$

A 95% confidence level was used (*Z* = 1.96), with a margin of error (*d*) of 5%. Because no prior prevalence estimate for breast cancer awareness among young women in Jazan was available, a conservative prevalence (*p*) of 0.50 was applied to ensure maximum sample size precision. Based on these parameters, the minimum required sample size was calculated as 384 participants. To account for potential nonresponse and to increase statistical power, the sample was increased to 480 women, which meets and exceeds the recommended threshold.

### 2.3 Study Measures and Questionnaire Development

The study measures involved a comprehensive approach to gathering data pertinent to breast cancer awareness and preventive behaviors. All items in the questionnaire were developed following a thorough examination of the literature pertaining to breast cancer research, including knowledge, behavior, and practices [22, 23, 24, 25, 26, 27, 28, 29]. The questionnaire was designed to capture a broad spectrum of variables relevant to breast cancer risk factors, personal health practices, and educational components.

Key demographic details such as gender, age, highest level of education, specialization, nationality, site type, employment status, marital status, number of children, and current health status were collected. Participants were also asked about their family history of breast cancer and personal health practices, including regular self‐examinations and medical screenings. The questionnaire further explored potential risk factors, including genetic predispositions, lifestyle choices, environmental exposures, hormonal influences, and stress. Awareness and educational aspects were assessed through questions on symptoms, genetic factors, and screening methods. Additional items regarding self‐examination frequency, confidence, and preferred educational resources ensured a robust evaluation of behavioral patterns.

In developing the questionnaire, 12 knowledge items were adapted from validated breast cancer awareness tools, including the Breast Cancer Awareness Measure [30, 31, 32]. Additionally, eight attitude items were adopted from instruments used in Middle Eastern breast cancer screening studies [6, 7, 15, 22], and six practice items were adapted from research assessing BSE and screening behaviors. A further seven context‐specific items were newly developed to reflect cultural, educational, and healthcare‐related factors relevant to women in the Jazan region. All adapted items underwent linguistic review to ensure cultural appropriateness and clarity.

### 2.4 Data Collection Procedures

The data were acquired through a Google survey link, downloaded as a CSV file, and imported into SPSS version 21. No missing or erroneous entries were identified due to complete response rates. Coding was developed for all questionnaire variables. Descriptive statistics included percentages, frequencies, means, and standard deviations.

### 2.5 Statistical Analysis

All statistical analyses followed the Statistical Analyses and Methods in the Published Literature (SAMPL) guidelines [33] and were performed using IBM SPSS Statistics, Version 29.0 (IBM Corp., Armonk, NY, USA). Descriptive statistics were reported as frequencies and percentages for categorical variables and as means with standard deviations for continuous variables. Independent‐samples *t* tests and one‐way ANOVA were used to compare mean knowledge, attitude, and practice scores across demographic groups; these were prespecified analyses, whereas subgroup comparisons exploring behavioral factors (e.g., self‐examination behavior) were considered exploratory. Effect sizes (correlation coefficients or odds ratios [ORs]) were presented alongside confidence intervals (CIs) to provide precision of estimates. Associations between continuous variables were evaluated using Pearson's correlation coefficients. Binary logistic regression was applied to identify predictors of BSE, and both crude and adjusted ORs with 95% CIs were reported. All statistical tests were two‐sided, and a priori significance was set at *α* = 0.05. Common statistical terms and abbreviations were defined at first use (e.g., OR = odds ratio, CI = confidence interval). Normality tests were run before statistical analysis.

### 2.6 Ethical Approval and Informed Consent

This study was reviewed and approved by the Jazan Health Ethics Committee, Ministry of Health, Kingdom of Saudi Arabia (Approval No. 23106). All procedures involving human participants adhered to institutional and national ethical standards and the Declaration of Helsinki. Informed consent was obtained from all participants.

### 2.7 Pilot Study

A pilot study was conducted among 30 women to evaluate the clarity, validity, and reliability of the questionnaire. Content validity was assessed by a panel of experts in public health and oncology, who confirmed the appropriateness and relevance of all items. Reliability testing demonstrated satisfactory internal consistency, with Cronbach's *α* values exceeding 0.70 across all domains (knowledge, attitude, and practice). Participants reported that the questions were clear, culturally appropriate, and easy to understand, and no major modifications were required. Data from the pilot study were excluded from the final analysis.

## 3 Results

Table 1 provides a summary of the demographic and health‐related variables, highlighting the major categories of each variable. The majority of respondents (58.1%) were < 25 years old, holding a bachelor's degree (57.5%) and specializing in health or medical specialties (42.8%). Nearly all respondents (97.1%) were from Saudi Arabia and most resided in urban areas (56.0%). Over half (56.5%) were students, and the majority were single (never married) (55.6%) with no children (62.5%). In terms of health, 86.5% reported having never been diagnosed with breast cancer and only 10% had a family history of breast cancer. Most respondents (61.7%) did not perform regular self‐examinations, 79.8% did not undergo medical breast cancer screenings, and only 8.1% underwent screenings regularly. This table offers a comprehensive overview of the respondents' demographic and health‐related characteristics, focusing on the major categories for each variable.

**Table 1:** Comparison of knowledge, attitude, and practice scores across demographic and behavioral characteristics.

Variable	Category description	*N*	%	Mean ± standard deviation		
				Knowledge score	Attitude score	Practice score
Age category						
	< 25 years	279	58.10	3.31 ± 0.66	4.02 ± 0.61	3.65 ± 0.72
	26–35 years	68	14.20	3.68 ± 0.68	4.14 ± 0.58	3.79 ± 0.72
	36–45 years	99	20.60	3.49 ± 0.55	3.97 ± 0.57	3.83 ± 0.63
	More than 45	34	7.10	3.72 ± 0.67	4.09 ± 0.58	3.84 ± 0.74
Highest level of education						
	Less than high school	26	5.40	3.31 ± 0.66	4.02 ± 0.61	3.65 ± 0.72
	High school or equivalent	136	28.30	3.68 ± 0.68	4.14 ± 0.58	3.79 ± 0.72
	Diploma/associate degree or equivalent	28	5.80	3.49 ± 0.55	3.97 ± 0.57	3.83 ± 0.63
	Bachelor's degree	276	57.50	3.72 ± 0.67	4.09 ± 0.58	3.84 ± 0.74
	Postgraduate	14	2.90	3.66 ± 0.42	4.02 ± 0.46	3.81 ± 0.65
Specialization						
	Health or medical specialties	136	42.80	3.78 ± 0.66	4.10 ± 0.59	3.83 ± 0.73
	Nonhealth specialization	344	71.70	3.62 ± 0.66	4.09 ± 0.58	3.81 ± 0.73
Nationality						
	Saudi Arabia	466	97.10	3.66 ± 0.67	4.09 ± 0.58	3.81 ± 0.73
	Others	14	2.90	3.80 ± 0.47	4.02 ± 0.54	4.12 ± 0.56
Current site type						
	Urban areas	269	56.00	3.64 ± 0.70	4.07 ± 0.63	3.82 ± 0.76
	Industrial areas	18	3.80	3.67 ± 0.69	4.17 ± 0.50	3.93 ± 0.60
	Rural areas	193	40.20	3.71 ± 0.61	4.12 ± 0.52	3.80 ± 0.69
Current employment status						
	Student	271	56.50	3.72 ± 0.71	4.12 ± 0.63	3.83 ± 0.77
	Housewife	76	15.80	3.59 ± 0.66	4.04 ± 0.55	3.76 ± 0.69
	Free works	6	1.30	3.28 ± 0.41	4.26 ± 0.29	3.89 ± 0.70
	Unemployed	23	4.80	3.55 ± 0.50	4.11 ± 0.51	3.80 ± 0.57
	Employee (part‐time or full‐time)	92	19.20	3.66 ± 0.58	4.07 ± 0.48	3.81 ± 0.68
	Retired	12	2.50	3.41 ± 0.38	3.89 ± 0.49	3.74 ± 0.68
Marital status						
	Single (never married)	267	55.60	3.70 ± 0.68	4.07 ± 0.61	3.79 ± 0.74
	Married	193	40.20	3.63 ± 0.64	4.10 ± 0.55	3.84 ± 0.70
	Divorced	11	2.30	3.68 ± 0.76	4.41 ± 0.48	3.98 ± 0.85
	Separated	5	1.00	3.78 ± 0.72	4.05 ± 0.69	4.23 ± 0.78
	Widowed	4	0.80	3.63 ± 0.53	3.95 ± 0.30	3.42 ± 0.40
Number of children						
	None	300	62.50	3.69 ± 0.70	4.09 ± 0.61	3.81 ± 0.75
	1	28	5.80	3.84 ± 0.65	4.31 ± 0.52	4.03 ± 0.76
	2	28	5.80	3.59 ± 0.45	4.15 ± 0.40	3.84 ± 0.57
	3	35	7.30	3.58 ± 0.59	4.03 ± 0.56	3.80 ± 0.53
	4+ (combined for simplicity)	89	18.50	3.60 ± 0.64	4.02 ± 0.52	3.77 ± 0.73
Current health status						
	I have never been medically diagnosed with breast cancer (free from breast cancer).	415	86.5	3.71 ± 0.63	4.12 ± 0.56	3.83 ± 0.70
	I am recovered from breast cancer.	65	13.5	3.43 ± 0.84	3.92 ± 0.69	3.74 ± 0.85
Family history of breast cancer						
	Yes	48	10	3.88 ± 0.68	4.22 ± 0.51	4.05 ± 0.62
	No	432	90	3.64 ± 0.66	4.08 ± 0.59	3.79 ± 0.73
Self‐examination behaviour						
	Yes	62	12.9	3.72 ± 0.70	4.12 ± 0.66	3.91 ± 0.67
	No	296	61.7	3.63 ± 0.67	4.05 ± 0.58	3.76 ± 0.74
	I perform self‐examinations only when there is some pain.	122	25.4	3.73 ± 0.63	4.18 ± 0.53	3.91 ± 0.70
Screening behaviour						
	Yes	39	8.1	3.69 ± 0.72	4.05 ± 0.70	3.87 ± 0.79
	No	383	79.8	3.66 ± 0.64	4.08 ± 0.57	3.76 ± 0.72
	I perform screenings only when there is some pain.	58	12.1	3.73 ± 0.75	4.19 ± 0.53	4.11 ± 0.67
	Total	480	100			

The analysis revealed several notable differences in breast cancer–related knowledge, attitudes, and practices across demographic and behavioral groups. Participants younger than 25 years generally demonstrated lower knowledge scores compared to older age groups, indicating a meaningful age‐related gap in awareness. Individuals in health or medical specialties showed higher knowledge scores than those in nonhealth fields, reflecting the influence of academic background on awareness levels. Participants without a prior breast cancer diagnosis had higher knowledge and attitude scores compared to those who had recovered from the disease. Likewise, those with a family history of breast cancer demonstrated higher knowledge and practice scores than participants without such a history, suggesting increased vigilance among high‐risk groups. Moreover, women who reported undergoing breast cancer screening exhibited higher practice scores than those who did not, underscoring the behavioral impact of screening engagement. Overall, these findings highlight age, academic specialization, health history, family history, and screening behavior as important determinants of breast cancer knowledge, attitudes, and preventive practices.

Participants' knowledge regarding various risk factors, symptoms, and screening methods related to breast cancer is presented in Table 2. The majority of participants agreed or strongly agreed that genetic factors (76.1%), lifestyle choices (81.6%), and environmental exposures (75.0%) contributed to breast cancer risk. Awareness of hormonal factors was slightly lower, with 71.2% acknowledging their impact. Misconceptions were evident, particularly regarding the regular use of aluminum‐containing deodorants, where 50.7% of participants remained neutral or agreed. Factors such as late pregnancy (after the age of 30 years) and not breastfeeding had lower agreement levels (28.1% and 43.4%, respectively). A strong awareness was observed regarding the common symptoms of breast cancer (81.5%) and the fact that men can also develop breast cancer (69.4%). However, knowledge of genetic predisposition (e.g., BRCA1/BRCA2 mutations) was moderate, with only 54.8% acknowledging their role. Awareness of medical screening methods, including mammography, MRI, and ultrasound, was relatively high (77.1%). Similarly, 61.5% reported knowing the correct method for self‐examination, while 51.6% understood the recommended frequency of breast cancer screening. Overall, the mean knowledge score across participants was 3.66 ± 0.66, indicating a moderate level of knowledge, with variations in awareness across different aspects of breast cancer risk and prevention.

**Table 2:** Participants' knowledge of breast cancer risk factors, symptoms, and screening methods.

Preferences	*N* (%)				
	Strongly disagree	Disagree	Neutral	Agree	Strongly agree
Genetic factors, such as a family history of breast cancer.	35 (7.3)	28 (5.8)	52 (10.8)	165 (34.4)	200 (41.7)
Lifestyle factors such as diet, exercise, smoking, and alcohol consumption.	21 (4.4)	19 (4.0)	48 (10.0)	197 (41.0)	195 (40.6)
Exposure to certain environmental toxins and chemicals.	32 (6.7)	28 (5.8)	60 (12.5)	194 (40.4)	166 (34.6)
Hormonal factors, including the use of certain birth control pills or hormone replacement therapy.	30 (6.3)	26 (5.4)	82 (17.1)	196 (40.8)	146 (30.4)
Regular use of deodorants containing aluminum.	27 (5.6)	49 (10.2)	161 (33.5)	153 (31.9)	90 (18.8)
Having the first pregnancy after the age of 30.	49 (10.2)	109 (22.7)	187 (39.0)	88 (18.3)	47 (9.8)
Not breastfeeding.	64 (13.3)	64 (13.3)	144 (30.0)	128 (26.7)	80 (16.7)
Frequent exposure to radiation, such as medical X‐rays.	32 (6.7)	27 (5.6)	101 (21.0)	169 (35.2)	151 (31.5)
Irregular physical activity.	44 (9.2)	89 (18.5)	158 (32.9)	116 (24.2)	73 (15.2)
Stress and psychological factors (contribute to the development of breast cancer).	19 (4.0)	28 (5.8)	121 (25.2)	184 (38.3)	128 (26.7)
I am aware of the common symptoms of breast cancer (e.g., lumps, changes in size or shape of the breast).	11 (2.3)	20 (4.2)	58 (12.1)	204 (42.5)	187 (39.0)
I recognize that men can get breast cancer, though it is less common than in women.	12 (2.5)	47 (9.8)	88 (18.3)	162 (33.8)	171 (35.6)
I am aware of the genetic aspects of breast cancer (e.g., BRCA1/BRCA2 gene mutations).	32 (6.7)	79 (16.5)	106 (22.1)	152 (31.7)	111 (23.1)
I am aware of the different medical screening methods (e.g., mammography, MRI, ultrasound).	11 (2.3)	31 (6.5)	68 (14.2)	222 (46.3)	148 (30.8)
I know the proper method for self‐examination.	28 (5.8)	57 (11.9)	100 (20.8)	179 (37.3)	116 (24.2)
I know when and how often breast cancer screenings are recommended.	24 (5.0)	103 (21.5)	105 (21.9)	147 (30.6)	101 (21.0)
Knowledge score (mean ± standard deviation)	3.66 ± 0.66				

Participants' attitudes toward breast cancer screening and early detection in the Jazan region, KSA, revealed strong acknowledgment of the importance of regular medical screenings and self‐examinations, with 54.6% strongly agreeing that they are crucial for early detection (Table 3). Approximately 87.7% consulted a doctor if they noticed unusual changes, indicating proactive health behavior. However, confidence in self‐examinations varied, with 23.1% feeling confident. There is also a notable reliance on professional advice for screening, as 67.1% would only undergo screening if recommended by a doctor. This highlights the need for enhanced education and resources to boost confidence in self‐examinations and encourage more independent health‐monitoring practices.

**Table 3:** Participants' attitudes toward breast cancer screening, self‐examination, and early detection.

Preferences	*N* (%)				
	Strongly disagree	Disagree	Neutral	Agree	Strongly agree
Regular medical screenings are important for early detection.	6 (1.3)	13 (2.7)	34 (7.1)	165 (34.4)	262 (54.6)
Self‐examination once a month is important for early detection.	12 (2.5)	32 (6.7)	88 (18.3)	203 (42.3)	145 (30.2)
Early detection increases the likelihood of successful treatment.	7 (1.5)	8 (1.7)	34 (7.1)	133 (27.7)	298 (62.1)
Self‐examination is important for everyone (both male and female).	0.0 (0.00)	23 (4.8)	45 (9.4)	181 (37.7)	231 (48.1)
Regular medical check‐ups are important for everyone.	0.0 (0.00)	17 (3.5)	37 (7.7)	180 (37.5)	246 (51.3)
I prefer to do self‐examination over medical examination.	0.0 (0.00)	81 (16.9)	136 (28.3)	153 (31.9)	110 (22.9)
I feel confident in my ability to perform a self‐examination.	0.0 (0.00)	83 (17.3)	123 (25.6)	163 (34.0)	111 (23.1)
I will only undergo medical screening if my doctor asks me to.	0.0 (0.00)	78 (16.3)	78 (16.3)	186 (38.8)	138 (28.8)
I will educate myself if I notice any unusual changes.	19 (4.0)	19 (4.0)	43 (9.0)	190 (39.6)	228 (47.5)
I will consult a doctor or specialist if I notice any unusual changes.	12 (2.5)	12 (2.5)	47 (9.8)	188 (39.2)	233 (48.5)
I will only undergo a medical screening if there is a health practitioner (for women) or with a health practitioner (for men).	0.0 (0.00)	61 (12.7)	93 (19.4)	153 (31.9)	173 (36.0)
Attitude score (mean ± standard deviation)	4.09 ± 0.58				

Participants' practices regarding BSE and breast cancer awareness are shown in Table 4. The majority (69.8%) preferred reading articles, 76.7% watched social media content (76.7%), and 68.1% attended educational programmes (68.1%). Visiting a doctor was the favored method, with 77.5% agreeing or strongly agreeing. Virtual medical consultations were less preferred, with only 62.3% favoring them. Seeking advice from friends or relatives had the lowest preference, with 48.1% neutral or disagree. These findings indicate that social media and professional healthcare consultations are key knowledge sources. The mean practice score was 3.81 ± 0.73, suggesting moderate engagement in learning behaviors.

**Table 4:** Participants' practices about breast self‐examination and breast cancer awareness.

Preferences	*N* (%)				
	Strongly disagree	Disagree	Neutral	Agree	Strongly agree
I prefer reading articles to learn about breast self‐examination.	18 (3.8)	36 (7.5)	91 (19.0)	199 (41.5)	136 (28.3)
I prefer watching social media content (YouTube, Snapchat, TikTok, etc.).	10 (2.1)	27 (5.6)	75 (15.6)	192 (40.0)	176 (36.7)
I prefer participating in educational programs or workshops (educational meetings or exhibitions).	14 (2.9)	32 (6.7)	107 (22.3)	179 (37.3)	148 (30.8)
I prefer virtual medical consultations (remote).	19 (4.0)	56 (11.7)	106 (22.1)	178 (37.1)	121 (25.2)
I prefer visiting the doctor to learn.	9 (1.9)	23 (4.8)	76 (15.8)	221 (46.0)	151 (31.5)
I prefer asking for advice from friends or relatives.	37 (7.7)	67 (14.0)	114 (23.8)	155 (32.3)	107 (22.3)
Practice score (mean ± standard deviation)	3.81 ± 0.73				

The correlations between knowledge, attitude, and practice scores related to breast cancer awareness showed clear and meaningful associations (Table 5). Knowledge demonstrated moderate positive correlations with both attitude (*r* = 0.569) and practice (*r* = 0.567), while the association between attitude and practice was stronger (*r* = 0.695), indicating that more positive attitudes were closely linked to better preventive behaviors. The reliability analysis showed good internal consistency across all scales, with Cronbach's *α* values ranging from 0.785 to 0.880. The mean scores for knowledge (3.66 ± 0.66), attitude (4.09 ± 0.58), and practice (3.81 ± 0.73) further reflected generally favorable levels of awareness and engagement among participants.

**Table 5:** Correlation between knowledge, attitude, and practice scores with reliability analysis.

Scores	Knowledge score	Attitude score	Practice score	Cronbach's *α*	Mean ± standard deviation
Knowledge score	1	0.569**	0.567**	0.880	3.66 ± 0.66
Attitude score	0.569**	1	0.695**	0.857	4.09 ± 0.58
Practice score	0.567**	0.695**	1	0.785	3.81 ± 0.73
*p* (two‐tailed)	0.000	0.000	0.000		
*N*	480				

** *p* < 0.01.

Table 6 presents the logistic regression analysis examining the factors associated with BSE, with ORs reported for both the crude and adjusted models. Several factors were significantly associated with BSE. Retired individuals had significantly lower odds of undergoing BSE (crude OR 0.195, *p* < 0.05). Having two or more children was associated with significantly reduced odds of BSE (adjusted ORs: 0.051, 0.065, and 0.054, *p* < 0.05). Older age groups (36–45 and more than 45 years) had reduced odds compared with younger individuals. Non‐Saudi participants had significantly lower odds of practicing BSE (crude OR odds ratio [OR] = 0.212, *p* < 0.05). The knowledge score was also significantly associated with BSE, indicating that higher knowledge levels were correlated with greater engagement in self‐examinations (crude OR 0.626, *p* < 0.05). Other factors, including educational level, employment status, and marital status, did not show strong associations in the adjusted model. These findings suggest that demographic and knowledge‐related factors influence BSE behavior.

**Table 6:** Logistic regression analysis of factors associated with breast self‐examination (BSE).

Factors	Categories	Crude OR	95% CI		Adjusted OR	95% CI	
			Lower	Lower		Lower	Upper
Current employment status	Student (reference)						
	Housewife	1.156	0.415	3.221	1.259	0.167	9.485
	Free works	0.000	0.000	0.00	00.00	0.000	0.00
	Unemployed	0.469	0.119	1.850	0.255	0.043	1.522
	Employee	0.553	0.262	1.166	0.426	0.057	3.173
	Retired	0.195*	0.049	0.777	0.246	0.015	4.034
Kids2	None (reference)						
	One child	0.74	0.20	2.72	0.383	0.041	3.537
	Two children	0.39*	0.13	1.18	0.051*	0.005	0.573
	Three children	0.49	0.15	1.60	0.065*	0.005	0.765
	More than 4	0.33*	0.15	0.69	0.054*	0.005	0.581
Highest level of education	Less than high school (reference)						
	High school or equivalent	2.045	0.486	8.608	1.654	0.197	13.861
	Diploma	00.545	0.095	3.146	0.472	0.036	6.123
	Bachelor's degree	1.425	0.376	5.406	1.144	0.136	9.651
	Postgraduate	0.682	0.085	5.448	0.782	0.048	12.677
Marital status	Single (reference)						
	Married						
	Divorced	0.583	0.307	1.109	1.968	0.314	12.326
	Separated	0.226*	0.050	1.014	0.481	0.056	4.170
	Widowed	0.271	0.024	3.119	0.422	0.019	9.214
Age	< 25 years (reference)						
	26–35 years	1.71	0.57	5.156	12.422	1.119	137.947
	36–45 years	0.70	0.32	1.511	6.941*	0.632	76.255
	More than 45	0.25*	0.09	0.67	4.709	0.262	84.658
Current site type	Urban areas (reference)						
	Industrial areas	.748	0.148	3.776	1.711	0.160	18.239
	Rural areas	1.371	0.722	2.604	1.291	0.625	2.666
Nationality	Saudi Arabia (reference)						
	Others	0.212*	0.062	0.723	0.410	0.086	1.957
Specialization	Health (reference)						
	Nonhealth college	0.987	0.510	1.910	1.345	0.537	3.367
Is there a family history of breast cancer?	Yes						
	No	0.795	.294	2.151	0.550	0.165	1.831
Knowledge score		0.626*	0.379	10.03	0.758	0.369	1.556
Attitude score		0.623	0.352	10.10	0.713	0.282	1.800
Practice score		0.629	0.404	0.98	0.838	0.410	1.713

* Significant at *p* < 0.05.

Women with 2+ children had significantly lower odds of performing BSE (AOR = 0.051–0.065, *p* < 0.05), suggesting that caregiving responsibilities hindered self‐care. Older women (36–45 years) showed increased BSE practices in the adjusted model (AOR = 6.941, *p* < 0.05), contrary to the crude model. Non‐Saudis initially had lower odds ratios (COR = 0.212, *p* < 0.05), but the effect diminished after adjustment (AOR = 0.410), indicating healthcare access disparities. While knowledge was significant in COR (0.626, *p* < 0.05), it lost significance in AOR, suggesting that awareness alone is insufficient and highlighting the need for practical interventions.

## 4 Discussion

The current study aimed to explore the role of demographic factors in breast cancer awareness and screening practices among younger women during the first two decades of their adult life in the Jazan Region of Saudi Arabia. Specifically, this study examined how age, educational attainment, health specialization, and other demographic factors act as proxies for the factors that determine the knowledge, attitude, and practice of breast cancer. This stress enables us to understand the impact of population segmentation on breast cancer preventive measures, and the barriers or facilitators that could lead to increased effectiveness in responding to the problem of late diagnosis.

The findings from this study reveal that demographic factors have an overwhelming impact on the awareness of breast cancer, which is in line with other studies [34, 35, 36, 37] where young females tend to be less active in breast cancer screening, which explains why those under 25 scored low in our knowledge assessment. For instance, the fact that health professionals scored higher in knowledge assessments parallels the result obtained by Rehman and colleagues, which highlighted how formal medical education improves disease‐related knowledge and health‐seeking behavior [38]. Additionally, the great proportionality that was found between the individual's and their family's health history and breast cancer awareness and practices has been further confirmed in a previous study that showed that people with a family history of breast cancer are more likely to use prevention and screening techniques [39]; thus, it appears that the experience or the genes in themselves make people more health‐conscious. Finally, the outcome of our study on screening behaviors having a positive impact on practice scores agrees with that of Smith and colleagues, where regular attendance at health preventive check‐ups was associated with better health behavior because of better health education and more contact with health professionals [40]. These findings collectively emphasize the need for multilayered public health policies that consider population demographics for the maximization of breast cancer education and prevention approaches worldwide.

The significant positive correlations found between knowledge, attitude, and practice scores in this study point toward a key relationship in health behavior theory, whereby greater education about breast cancer contributes to enhanced prevention and treatment‐seeking behavior. The moderate correlation between knowledge and attitude (*r* = 0.569) suggests that improved perception is associated with more knowledge toward breast cancer prevention, thus supporting the view that educational interventions have the potential to change attitudes, which Carpenter identified as the first step in changing behavior [41]. The correlation between knowledge and practice (*r* = 0.567) also suggests that individuals who are more knowledgeable are more willing to adopt certain practices, such as participating in regular screening tests, because the Health Belief Model claims that knowledge of a disease‐negative outcome encourages people to take protective action [42]. In addition, the high correlation of attitude and practice (*r* = 0.695) indicates that attitudes rather than knowledge are more important in determining the level of engagement in preventive practices. As documented by Albarracín and colleagues, attitudes are mediators of the relationship between knowledge and behavior [43]. Such resources are relevant for developing all‐embracing educational programs that not only give pass information but also seek to positively influence attitudes toward increased compliance to recommended practices and enhanced health results.

Discussing the factors related to BSE entails the analysis of several essential predictors that warrant concern. First, retirement is a crucial demographic characteristic that comes to the line; retirees are less likely to perform BSE compared to those employed, which is very significant. This is probably due to a reduction in health‐seeking behavior, as noted by Syse et al. for the aged population, which is evident after the age 22 [44]. Similarly, having two or more children limits the chances of BSE being performed because parental obligations may supplant personal health activities. This notion was corroborated by a previous study that showed the dominance of caregiving activities over personal healthcare services [45].

A potential limitation of this study is that it is self‐reported, which may lead to bias, as participants may try to answer in a way in which they are more socially accepted. To combat this, future studies may attempt to integrate clinical records, which are more objective measures of self‐reported health behaviors. Another limitation is the use of a cross‐sectional design, as this type of study does not allow one to infer the causation of factors in BSE practices. There is evidence that behavioral changes occur over time, which means that longitudinal studies would be more appropriate for carrying out these investigations. Furthermore, those making the study may be based on their research within a narrow geographic and cultural scope, thus limiting the applicability of the results. The inclusion of other groups in this research may improve external validity.

## 5 Conclusions

This research sheds light on the fundamental demographic and awareness‐related factors that impact women's BSE performance in Jazan, Saudi Arabia. The results showed that the influence of BSE activities is age‐dependent, as is the relationship between employers and those practicing BSE, including those with familial links to breast carcinomas. Additionally, having a higher knowledge score on BSE lowers the odds of practicing BSE, emphasizing the importance of education in health practice. These findings are essential for public health policy because education can improve behavioral change toward BSE practices among younger, uninformed women from different cultures. Future intervention recommendations involve preparing group‐specific educational campaigns to overcome barriers to these subgroups, such as retired and foreigners. Furthermore, BSE should be incorporated into other health and wellness programs to boost participation and early detection, eventually improving breast cancer results.

## Author Contributions

All authors made a significant contribution to the work reported, whether in the conception, study design, execution, acquisition of data, analysis, and interpretation, or in all these areas. All authors participated in drafting, revising, or critically reviewing the article, gave final approval of the version to be published, agreed on the journal to which the article has been submitted, and agreed to be responsible for all aspects of the work. The corresponding author, Siddig Ibrahim Abdelwahab (S.I.A.), had full access to all study data and assumes complete responsibility for the integrity and accuracy of the data analysis. All authors have read and approved the final version of the manuscript.

## Funding

The authors gratefully acknowledge the funding of the Deanship of Graduate Studies and Scientific Research, Jazan University, Saudi Arabia, through Project Number: (JU‐202503204‐DGSSR‐RP‐2025). The funding body *had no role* in the study design; data collection, analysis, or interpretation; writing of the manuscript; or the decision to submit this work for publication.

## Disclosure

The lead author Siddig Ibrahim Abdelwahab affirms that this manuscript is an honest, accurate, and transparent account of the study being reported; that no important aspects of the study have been omitted; and that any discrepancies from the study as planned (and, if relevant, registered) have been explained.

## Ethics Statement

This study was reviewed and approved by the Research Ethics Committee of Jazan University, Kingdom of Saudi Arabia (Approval No. 1485). All procedures performed in this study involving human participants were in accordance with the Ethical Standards of the Institutional and/or National Research Committee and with the 1964 Helsinki Declaration and its later amendments or comparable ethical standards.

## Consent

Informed consent was obtained from all individual participants included in the study.

## Conflicts of Interest

The authors declare no conflicts of interest.

## Data Availability

The authors confirm that the data supporting the findings of this study are available from the corresponding author, upon reasonable request. No publicly archived data sets are associated with this article due to participant confidentiality restrictions.
